# Development of Late Toxicities in Patients with Oral Tongue Cancer Treated with Surgical Resection and Adjuvant Radiation Therapy

**DOI:** 10.3389/fonc.2016.00272

**Published:** 2017-01-11

**Authors:** Mutlay Sayan, Richard J. Cassidy, Jeffrey M. Switchenko, Oluwatosin A. Kayode, Nabil F. Saba, Conor E. Steuer, Dong M. Shin, J. Trad Wadsworth, Mark El-Deiry, Mihir Patel, Jonathan J. Beitler, Kristin A. Higgins

**Affiliations:** ^1^University of Vermont Medical Center, Burlington, VT, USA; ^2^Department of Radiation Oncology, Emory University, Atlanta, GA, USA; ^3^Winship Cancer Institute, Emory University, Atlanta, GA, USA; ^4^Department of Biostatistics and Bioinformatics Shared Resource, Emory University, Atlanta, GA, USA; ^5^Department of Hematology and Medical Oncology, Emory University, Atlanta, GA, USA; ^6^Department of Otolaryngology – Head and Neck Surgery, Emory University, Atlanta, GA, USA

**Keywords:** oral tongue cancer, radiation therapy, PEG tube dependency, osteoradionecrosis, narcotic dependency

## Abstract

**Objectives:**

The late effects of RT are not well reported in patients with oral tongue cancer (OTC). This study reports the incidence of late effects and factors associated with the development of late effects in OTC patients.

**Methods:**

Patients with OTC treated in our institution from 2003 to 2013 were evaluated. The association between RT doses, including mandible maximum and minimum doses and total 3D maximum dose, and late toxicity, defined as development of osteoradionecrosis (ORN), percutaneous endoscopic gastrostomy (PEG) tube dependence for >6 months after treatment, and narcotic dependency >6 months posttreatment were assessed using both univariate and multivariable (MV) analysis.

**Results:**

Seventy-six patients with OTC (45% males and 55% females) were treated with definitive surgical resection followed by adjuvant RT. The median follow-up was 4.3 years. Combined late toxicities were reported in 38% of patients. Thirty-four percent of the patients had narcotic dependency and, 3.9% of the patients had ORN of the mandible. Thirteen percent of patients developed PEG tube dependency that was significantly associated with a higher 3D maximum radiation dose on univariate analysis (*p* < 0.01). On MV analysis, 3D maximum dose remained significantly associated with long-term PEG tube dependency (*p* = 0.05).

**Conclusion:**

Patients with OTC treated with adjuvant RT are at significant risk for development of late toxicities. Increasing maximum dose is associated with long-term PEG tube dependence, and care should be taken to reduce the “hot spot” within radiation treatment plans as much as possible.

## Introduction

Cancers of the oral tongue represent the most common primary site of oral cavity cancer (OCC), the majority of which are squamous cell carcinomas. In 2014, approximately 28,030 patients were diagnosed with OCC, of which 13,590 had oral tongue cancer (OTC) (1). The current standard of care for OTC is surgical resection followed by adjuvant therapy depending on pathological characteristics of disease (2, 3). Notably, the survival benefit of elective neck dissection at the time of initial surgical management relative to salvage neck dissection at the time of nodal relapse was recently reported in a randomized control trial (4). After resection, the presence of adverse pathological features, specifically bone invasion, tumor thickness >4 mm, lymphovascular or perineural invasion (PNI), or multiple positive lymph nodes are indications for postoperative management with RT (5, 6). Surgical resection followed by concurrent chemotherapy and RT is recommended for patients with positive margins or lymph nodes with extracapsular extension (3, 7, 8).

Advances in reconstructive surgery have led to better functional outcomes following primary surgical resection (9). RT and concurrent, radiosensitizing chemotherapy continue to be integral components of the treatment paradigm to improve both locoregional control and survival; Radiation Therapy Oncology Group (RTOG) (8) and EORTC (7) trials showed 10% improvement in locoregional control in head and neck cancer patients treated with concurrent postoperative chemotherapy and radiotherapy relative to radiation therapy alone. These improvements in locoregional control, however, come at the expense of increased toxicities. In RTOG 9501 and EORTC 22931 trials, the incidence of grade ≥3 acute toxicity is approximately twice as high with concurrent treatment; however, grade 3 or higher late toxicities were similar among the groups in RTOG and EORTC trials, at approximately 30–40%.

Given such high rates of grade 3 or higher late toxicities in postoperative head and neck cancer patients, the goal of this study was to retrospectively analyze clinical and treatment-related variables that may contribute to the development of late side effects within OTC patients. Specific late toxicity endpoints in this study include osteoradionecrosis (ORN) of the mandible, long-term percutaneous endoscopic gastrostomy (PEG) tube dependence, and long-term narcotic dependency.

## Materials and Methods

### Study Population

After Institutional Review Board (IRB) approval at the Winship Cancer Institute of Emory University, a retrospective chart review of OTC patients treated between 2003 and 2013 was performed (IRB code number: 00016211). Inclusion criteria for this study included a confirmed diagnosis of squamous cell carcinoma of the oral tongue and surgical resection followed by RT delivered at Winship Cancer Institute. Patients treated by surgical resection alone, patients receiving adjuvant treatment outside of Emory, and patients with any distant metastases at the time of diagnosis were excluded from this study. All patients had routine pretreatment evaluations consisting of a complete history, physical examination, blood tests, computed tomography (CT), or positron emission tomography (PET) of the head and neck region and chest X-ray or chest CT as clinically appropriate.

### Data Collection

All data were collected using electronic medical records and departmental radiation oncology charts. The clinical parameters of interest included tumor characteristics, stage, age, radiation techniques and dose, treatment dates, and late toxicities following radiation. In this study, data for late toxicity including ORN, PEG tube placement, and narcotic dependency rates were retrieved from clinical notes at each follow-up visit. All radiological examination reports were reviewed for general pathological changes and for changes specific to ORN lesions. PEG tube dependency was reported as continuation of tube feedings 6 months after completion of treatment and narcotic dependency as continued use after 6 months of completion of treatment.

### Radiotherapy Planning and Delivery

Computed tomography simulation was completed following the surgery using a GE Lightspeed Large Bore CT Scanner (GE Healthcare, Waukesha, WI, USA). A thermoplastic head and shoulder mask (WFR/Aquaplast Corporation, Wyckoff, NJ, USA) was used for each patient during simulation and treatment for immobilization. The treatment planning CT and intravenous contrast was matched with PET (if obtained) in the treatment position to better define target volumes. The RTOG guidelines were used to contour organs at risk (OARs) (10). Dose calculations were performed in Eclipse Treatment Planning System v10.6 (Varian Medical Systems, Palo Alto, CA, USA) using the convolution/superposition algorithm to correct for tissue heterogeneities. Treatments were delivered by using a Varian Linear Accelerator (Varian Medical Systems, Palo Alto, CA, USA) with 6 MV photons with intensity-modulated radiation therapy (IMRT) or volumetric-modulated arc therapy techniques. Daily onboard imaging with KV X-rays was obtained prior to each treatment, with MV imaging utilized only if KV imaging was unavailable. Dosimetric variables were captured from eclipse planning software and recorded, including 3D maximum dose, prescribed total dose, and mandible minimum, maximum, and mean doses.

### Statistical Analysis

Statistical analysis was performed using SAS 9.4 statistical software (SAS Institute Inc., Cary, NC, USA). Categorical variables were compared across toxicity endpoints using chi-squared tests or Fisher’s exact tests, where appropriate, while continuous variables were compared across endpoints using ANOVA. For the multivariable (MV) logistic regression models, we modeled the probability of an event (long-term narcotic dependence, PEG tube dependence, or any adverse event) as a function of 3D maximum dose (%) and the three most significant predictors in each univariate comparison. For ORN, we modeled the probability of ORN as a function of mandible minimum dose (%) and mandible maximum dose (%). The number of variables in each MV model was restricted due to the low number of toxicity events.

## Results

### Patient and Treatment Characteristics

Seventy six patients (45% males and 55% females) met the inclusion criteria. The median follow-up time was 4.3 years. Patient characteristics are demonstrated in Table 1. Median age at diagnosis was 56 years (range, 15–89 years). Squamous cell carcinoma was the histology type among all patients. PNI was present in 55% of the patients and angiolymphatic invasion in 30%. Three (4%) patients had a bone invasion. Margins were positive in 7% of the patients and <5 mm in 30%. Moderately differentiated tumor was the most prevalent, reported in 58% of the patients.

**Table 1:** **Patient characteristics**.

Characteristics	No. of patients (%)
Age (y)	
Median	56
Range	15–89
Race	
White	59 (78)
Other	17 (22)
Gender	
Female	42 (55)
Male	34 (45)
Histology	
SCC	76 (100)
T stage	
T1	35 (46)
T2	21 (28)
T3	8 (10)
T4	12 (16)
N stage	
N0	42 (55)
N1	19 (25)
N2	15 (20)
Bone invasion	3 (4)
PNI	42 (55)
ALI	23 (30)

*SCC, squamous cell carcinoma; PNI, perineural invasion; ALI, angiolymphatic invasion; y, year*.

Table 2 summarizes the treatment characteristics. Thirty-three percent of the patients received chemotherapy. Cisplatin and carboplatin/paclitaxel were the most frequently used regimens. Chemotherapy was administered concurrently with RT in all patients receiving chemotherapy. The primary tumor alone was resected without neck dissection in 9% of patients. Partial glossectomy plus unilateral neck dissection was performed in 91% of patients. Total glossectomy was performed on three patients. Radiation dose was between 59.4 and 70.29 Gy in 1.8–2.2 Gy per fraction in all patients (Table 2). The median 3D dose maximum was found to be 109% (range, 104–129%).

**Table 2:** **Treatment characteristics**.

	No. of patients (%)
Radiology	
CT	57 (75)
PET/CT	19 (25)
Chemotherapy	
None	51 (67)
Cisplatin	18 (24)
Carboplatin and paclitaxel	6 (8)
Cetuximab	1 (1)
Concurrent chemoradiotherapy	25 (33)
Surgical procedure	
Primary alone	7 (9)
Primary and ND	69 (91)
Radiation therapy	
Prescribed dose	
Median	63 Gy
Range	(59.4–70.29 Gy)
Fraction size	
Median	2 Gy
Range	(1.8–2.2 Gy)
3D dose max (%)	
Mean	109
Median	109
Range	(104–129)
Mandible min. dose	
Mean	3.5 Gy
Median	2 Gy
Range	(0–13 Gy)
Mandible max. dose	
Mean	63 Gy
Median	66 Gy
Range	(10–85 Gy)
Mandible median dose	
Mean	47 Gy
Median	50 Gy
Range	(8–76 Gy)

*ND, neck dissection; CT, computed tomography; PET, positron emission tomography*.

### Late Toxicity

Late toxicity was reported in 38% of patients. Three patients developed ORN alone or with other late toxicities, 10 patients had PEG tube dependency alone or with other late toxicities, and 26 patients had narcotic dependency alone or with other late toxicities (Table 3). Receipt of chemotherapy had no influence on the development of late toxicities (*p* = 0.439). Surgical procedures alone had no significant effect on reported late toxicities (*p* = 0.468). Median prescribed radiation dose was 63 Gy (range, 59.4–70.29) in patients with no toxicity and identical in patients who developed the late toxicities. Furthermore, there was no significant difference in fraction size (*p* = 0.643). There were no significant differences in mandibular median (*p* = 0.847), maximum (*p* = 0.334), or minimum (*p* = 0.834) doses in patients who developed late toxicities versus those who did not.

**Table 3:** **Late toxicity outcomes**.

	No. of patients with late toxicity	% of patients with late toxicity
ORN	3	3.9
PEG dependence at 6 mo	10	13
Narcotic dependency	26	34
Total patients with late toxicity	29	38

*ORN, osteoradionecrosis; PEG, percutaneous endoscopic gastrostomy; mo, month*.

Individual late toxicities—ORN, PEG tube dependency, and narcotic dependency—were then analyzed separately (Table 4). The 3D maximum dose (%) was statistically significant for long-term PEG tube dependency on univariate analysis (*p* = 0.002). On MV analysis, the 3D maximum dose (%) was significantly associated with long-term PEG tube dependency (Table 5).

**Table 4:** **Univariate analysis of dosimetric parameters**.

Parameters	Yes	No	*p*
**PEG tube dependence**			
3D dose max (%)			0.002
Mean	112.5	108.8	
Median	110.7	108.7	
Number of patients	10	66	
**Narcotic dependence**			
3D dose max (%)			0.53
Mean	108.9	109.5	
Median	108.3	109.4	
Number of patients	26	50	
**Osteoradionecrosis**			
3D dose max (%)			0.12
Mean	106.0	109.4	
Median	106.9	109.2	
Number of patients	3	73	

*max, maximum; PEG, percutaneous endoscopic gastrostomy*.

**Table 5:** **Multivariable analysis for late toxicities**.

	Odds ratio (95% CI)	*p*
**Long-term PEG dependence**		
3D dose max	1.27 (1.00–1.61)	0.05
T stage (reference = T4)		
T1	0.46 (0.03–6.29)	0.08
T2	1.12 (0.12–9.97)	
T3	0.62 (0.03–13.45)	
Margins (reference ≤5 mm)		0.26
Positive	1.21 (0.09–16.84)	
Negative	0.26 (0.05–1.43)	
Radiology (reference = CT)		
PET/CT	1.88 (0.32–10.96)	0.48
**Osteoradionecrosis**		
Mandible min. dose (%)	0.87 (0.57–1.32)	0.51
Mandible max. dose (%)	0.97 (0.93–1.01)	0.18
**Long-term narcotic dependence**		
3D dose max (%)	0.97 (0.83–1.12)	0.65
PNI (reference = negative)	0.97 (0.83–1.12)	0.21
ALI (reference = negative)	0.61 (0.18–2.11)	0.44
Treatment group (reference = post-CRT)		0.66
Surgery	1.46 (0.33–6.55)	
Postoperative RT	0.83 (0.21–3.20)	

*CI, confidence interval; min, minimum; max, maximum; PET, positron emission tomography; CT, computed tomography; PNI, perineural invasion; ALI, angiolymphatic invasion; RT, radiation therapy; CRT, chemoradiation therapy*.

## Discussion

In the present study, we demonstrate that 38% of patients with OTC developed late toxicities (inclusive of long-term PEG dependence, ORN, and long-term narcotic dependence) after surgical resection and adjuvant radiation treatment. When analyzing all late toxicities together, there were no specific variables associated with the development of late toxicities. However, when analyzing individual late toxicities, the maximum radiation dose, or “hot spot,” was significantly higher in patients with long-term PEG tube dependency on both univariate and multivariate analysis.

Mitigation of toxicities and mechanisms to improve quality-of-life are areas of active research for patients with OTC. While the addition of chemotherapy to postoperative radiation improved 5-year overall survival rate from 40 to 53% in EORTC trial, it also resulted in increased toxicity (7). Aside from acute toxicity, treatment-associated adverse effects remain prominent even years after completion of therapy. Late onset dysphagia has been reported to remain unchanged in 48% of patients’ years after completion of therapy (11, 12). It is therefore clear that a better understanding of factors that drive late toxicities in this population is needed, in order to improve long-term function of OTC patients.

Designing radiation plans that limit dose heterogeneity and maximum dose is a critical part of the radiation treatment planning process. RTOG 0920, an active phase III trial of surgery and postoperative radiation ± cetuximab mandates a maximum dose (or hot spot) outside of the PTV to be limited to ≤110% of the prescribed dose. Keeping the maximum dose to ≤110% is ideal, as hot spots above 110% put normal tissue at risk for significant side effects of radiation. In order to minimize the maximum dose, or hot spot, dosimetrists create optimization planning target volumes (OPTI-PTV) and create avoidance structures for OARs. However, for some difficult cases where the initial optimization meets all given parameters but has a hot spot greater than 110%, the dosimetrist will convert an isodose line of less than 110% (between 106 and 108%) to a control point structure and reoptimize the plan starting from the last iteration and give a constraint of equivalent dose of the structure with a high priority. In the majority of cases this will minimize the hot spot below 110%. The dosimetrist can create another control point structure and repeat the same process or they can manipulate the normal tissue optimization settings and reoptimize the plan. That can be done alone or combined with the second control point structure. In most cases, these steps will drop the hot spot to less than 110%.

Prophylactic gastrostomy tube placement has been routinely offered to patients with locally advanced head and neck cancer prior to initiating definitive chemoradiation therapy at our institution. During treatment, patients are closely assessed by speech pathologists to determine swallowing function. After completion of therapy, PEG tube removal is recommended if patients are able to tolerate all nutrition orally for at least 2 weeks and adequately maintain weight. In the past, we have reported that 25% of elderly patients with oropharyngeal squamous cell carcinoma develop PEG tube dependency (13). In this study, 13% of patients developed PEG tube dependency. This is lower than cases reported in existing literature and our previous experience (14, 15). Inclusion of only OTC patients in our analysis may be a contributor for this result. Furthermore, non-adherence to swallowing exercises has been suggested to be related this late toxicity (16), even though compliance to swallowing exercises was not assessed, it is possible that potential higher adherence in our patient population may contribute to lower PEG tube dependency. Prior studies have also demonstrated that preoperative weight loss, heavy alcohol use, low socioeconomic status, and lack of social support (i.e., the absence of a partner) are also risk factors for the development of long-term PEG tube dependency (17–22).

Pain following head and neck cancer therapies often leads to significant psychological and physical suffering resulting in long-term narcotic dependency. Radiation-induced mucositis typically improves 4–6 weeks after completion of treatment (23); however, 50% of patients treated with RT with and without chemotherapy were unable to discontinue opioid use more than 3 months after completion of therapy, and 33% of patients required continued opioid use at 6 months (24). Here, we showed that 34% of patients continue to use narcotics 6 months after the completion of therapy. A large systematic review analysis showed that after completion of multimodality therapy, pain was increased in 33% of patients compared to the pretreatment baseline and unchanged in 36% of patients with locally advanced head and neck cancer (25). These previous studies did not clearly delineate subtypes of HNCs and reported results as a general group. In this study, we demonstrate that OTC patients have similar long-term narcotic dependency rates as compared to other subsites. While much of the increased pain has been associated with mucositis leading to the development of narcotic dependency, neither pain level nor previous history of substance abuse were assessed among these patients, which has been associated with development of narcotic dependency in patients with HNC (24).

In our study, 3.9% of patients developed ORN, within previous reports in the literature. The incidence of ORN was estimated to be 4.74–37.5% historically; however, less than 5% incidence has been reported in recent studies, likely attributed to the improvement of dental care and radiation techniques (26–31). No association between ORN and RT was demonstrated in our series, which is likely due to the low number of ORN events in this study. Furthermore, in addition to radiation dose, there are many other risk factors for developing ORN including oral hygiene, dental extractions, nutritional status, alcohol, and tobacco use (24, 30, 32, 33). Tobacco and alcohol use may directly cause ORN due to its effects on mucosal breakdown, but they are also surrogates for poor oral hygiene. Patients with poor oral hygiene are reported to have a 3.06 times greater odds of developing non-resolving ORN than patients with good oral hygiene (34).

There are several limitations of this study. First, the retrospective nature of this study hinders the ability to collect data on other relevant late toxicity endpoints for this patient population, including fibrosis and more detailed data regarding swallowing function and xerostomia. Additionally, other risk factors for developing late toxicities, including tobacco use, were not consistently reported and therefore were not included in this analysis. Second, the cohort included in this study included oral tongue patients only; therefore, the number of patients was small with a median follow-up time of 25.8 months. This small sample size is likely underpowered to detect potential significant differences. Furthermore, improvements in treatment planning techniques during the time period of this study could have also accounted for variation in treatment-related toxicity. There is certainly a learning curve with IMRT treatment planning; however, in order to generate a large cohort of oral tongue patients, the study time period is somewhat extensive and thus variability in treatment planning over time remains an issue. Larger prospective studies are required to overcome such limitations in order to provide improved insights into the development of late toxicities.

In conclusion, this study demonstrates that the risk of long-term narcotic dependence, ORN, or PEG tube dependence is 38% after completion of multimodality treatment for OTC. Furthermore, the development of long-term PEG tube dependency in these patients was significantly associated with higher RT maximum dose. Further evaluation of late toxicities in the randomized setting is needed in order to better understand patient-specific and treatment-related risk factors that may increase the likelihood of late side effects after treatment. Care should be taken to minimize the “hot spot” in radiation planning for patients with OTC treated postoperatively in order to reduce the risk of long-term PEG tube dependency.

## Author Contributions

MS, RC, JS, OK, NS, CS, DS, JW, ME-D, MP, JB, and KH all contributed to study concept, design, and/or acquisition of data. MS and RC completed the data collection. JW contributed to the data analysis. MS and KH were responsible for drafting the manuscript. All authors contributed to revising and giving final approval to the manuscript. All authors agree to be accountable for all aspects of the work including its accuracy and integrity.

## Conflict of Interest Statement

The authors declare that the research was conducted in the absence of any commercial or financial relationships that could be construed as a potential conflict of interest.
